# Development and Validation of a Field-Based Colorimetric LAMP Assay for the Detection of *Clavibacter michiganensis* in Tomato Plants

**DOI:** 10.3390/plants15030372

**Published:** 2026-01-25

**Authors:** Glykeria Mermigka, Maria Megariti, Dimitris Malliarakis, Marianthi G. Pagoulatou, Electra Gizeli, Dimitrios E. Goumas

**Affiliations:** 1 Laboratory of Biotechnological Applications and Plant Protection, Department of Agriculture, School of Agricultural Sciences, Hellenic Mediterranean University, 71410 Heraklion, Greece; dim-mal@hotmail.com (D.M.); mpagoulatou@hmu.gr (M.G.P.); 2 Institute of Agri-Food and Life Sciences, University Research Centre, Hellenic Mediterranean University, 71410 Heraklion, Greece; 3 Foundation for Research and Technology-Hellas, Institute of Molecular Biology and Biotechnology, 70013 Heraklion, Greece; maria_megariti@imbb.forth.gr (M.M.); gizeli@imbb.forth.gr (E.G.); 4 Department of Biology, University of Crete, 70013 Heraklion, Greece

**Keywords:** point-of-care diagnostics, LAMP, tomato, LoD, bacterial canker

## Abstract

Point-of-care diagnostics are revolutionizing the detection of plant pathogens by enabling rapid, on-site identification without the need for specialized laboratories. One of the tools used for this purpose is loop-mediated isothermal amplification (LAMP). LAMP is a powerful molecular technique increasingly used in pathogen control for its rapid, sensitive, and specific detection of plant pathogens. The aim of this study was the development of a novel, easy-to-use portable colorimetric LAMP (cLAMP) assay that could be used by inexperienced personnel for the detection of the pathogen *Clavibacter michiganensis*. The assay was combined with a newly constructed device in which LAMP can be performed in 30 min. Initially, a new set of LAMP primers targeting the *micA* gene was designed and evaluated the sensitivity (100 fg/reaction) and specificity of the assay. Next, the limit of detection (LoD) of two different commercial LAMP kits was compared with common laboratory detection techniques (DAS-ELISA, immunofluorescence, quantitative PCR, and PCR) using the same samples. Additionally, the LoD of the developed cLAMP assay was evaluated in bacterial suspensions and plant extracts spiked with *C. michiganensis* and validated the effect on the LoD of plant extracts from different tomato varieties. Lastly, its efficacy for *C. michiganensis* detection was assessed in experimentally inoculated tomato seedlings. The developed method for *C. michiganensis* detection can be used as a reliable tool for the early detection of the pathogen for field-based applications by untrained personnel.

## 1. Introduction

In agriculture, diagnostic assays are essential for increasing crop sustainability and productivity. Advanced technologies, including remote sensing, drones, and AI-powered imaging, enable farmers to monitor plant health in real time [1]. By integrating these diagnostic tools, modern agriculture can reduce resource waste, improve yields, adapt to challenges like climate change, and identify pests and diseases, thus ensuring food security for a growing global population. Concerning disease management, early, rapid, and accurate detection of the pathogen at the species/strain level is a crucial factor for preventing the disease spread, a task that is mainly conducted with the use of molecular tools [2]. Until recently, molecular diagnosis for plant disease detection was limited to centralized laboratories, which entail sample transportation from the collection site to the laboratory, the need for experienced personnel and proper equipment and, lastly, delay in result documentation. To overcome all these disadvantages, point-of-care (POC) diagnostic tests are increasing rapidly.

POC testing refers to those research and reference resources conducted at or near the sampling location, typically outside of a centralized laboratory setting. These diagnostics enable agricultural/medical professionals but also unspecialized personnel to obtain immediate results without relying on off-site laboratories, facilitating timely decision-making. Although their first report dates back to the early 1970s [3], POC testing has expanded quickly and globally in the last ten years [4]. The loop-mediated isothermal amplification (LAMP) assay is one of the several diagnostic methods used for plant pathogens detection [5].

LAMP, a method introduced almost 30 years ago [6], is a reliable and robust method for the detection of DNA in biological samples. It relies on the isothermal amplification of the DNA target molecule with the use of up to three primer pairs [7]. It is highly specific like polymerase chain reaction (PCR), but unlike PCR, LAMP relies on isothermal amplification. Due to its accuracy, ease of use, and speed in result documentation, LAMP has been widely used for the detection of plant pathogens not only in centralized laboratories but also as a POC diagnostic tool [8]. However, the latter entails thorough experimentations to evaluate a variety of crucial parameters such as specificity, limit of detection (LoD), and appropriate sampling method depending on the targeted pathogen.

*Clavibacter michiganensis* is a Gram-positive, aerobic bacterium and the causative agent of bacterial canker in tomatoes, leading to symptoms such as wilting, leaf spots, and fruit lesions, which can severely impact agricultural yields [9,10]. The bacterium spreads through contaminated seeds, tools, or water splashes, making it a challenge to control in farming nursery and filed environments. Especially, contaminated seed stocks contribute to the bacterium’s global spread, with one infected seed out of 10,000 having the potential to start an epidemic [11]. Since prevention through seed and seedling health control is currently the only method to combat this pathogen [11], it is crucial to identify it early in the event of infection, ideally before disease symptoms manifest in the crop [12].

For this purpose, a commercial portable device, the PEBBLE colorimetric LAMP (cLAMP) platform (BIOPIX-T) was used for POC diagnosis of the bacterium *C. michiganensis*. To develop and validate *C. michiganensis* detection for POC use with this commercial technology, a series of experiments was set up, which included primer validation, identification of its LoD in various samples (extracted DNA, bacterial suspensions, plant extracts), and a comparison of it with other commonly used methods. All the above experimentation was conducted with the goal of developing an easy-to-use process that could be performed by inexperienced personnel.

## 2. Materials and Methods

### 2.1. Bacterial Isolates and Culture Conditions

The bacterial isolates used in this study were *C. michiganensis* (CFBP4999^T^), *Pseudomonas syringae* pv. *tomato* (DC3000), and three species isolated from naturally infected plant samples: *Xanthomonas campestris* pv. *vesicatoria* (HMU8020) and *Bacillus* sp. (GL2), *Clavibacter sepedonicus* (HMU4075B and HMU4083A). The bacteria were cultivated at 26 °C for 48 h in nutrient agar medium (NA, Condolab, Madrid, Spain) supplemented with 0.5% (*w*/*v*) glucose. After that period, the inoculum was resuspended in distilled water (dH_2_O) and the optical density at 600 nm (OD_600_) was measured and adjusted depending on the experiment.

### 2.2. Plant Material and Growth Conditions

Tomato plants (*Solanum lycopersicum*) were placed in a growth chamber in a mixture of compost and perlite (2:1 ratio) in a 16 h light/8 h dark photoperiod at 25 °C. In most experiments, the cultivar “Elpida” was used while plant extracts from the cultivars Lobello, Kalloni, Ekstasi, Lesvos, Christina, Nissos, and Aethra were also used.

### 2.3. Plant Inoculation Assays

Seedlings of the tomato cultivar Elpida, at the stage of three true leaves, were gently punched with a needle on the stem right beneath the cotyledons. A total of 10 μL of *C. michiganensis* (Cm) inoculum corresponding to 3 × 10^6^ CFU/mL were carefully placed on the wound. Negative controls (healthy plants) were treated similarly as the infected plants except for placing 10 μL of dH_2_O on the wound. The plants were placed back in the growth chamber at 50–60% humidity.

### 2.4. Calculation of Colony Forming Units

To determine bacterial concentrations in various extracts or bacterial suspensions, successive dilutions were performed and 5 µL of each dilution was spotted in triplicates in NAG medium. The Petri dishes were incubated at 26 °C for 2–3 days. CFU per mL were calculated by counting the colonies and using the following formula: number of colonies × dilution factor/volume spotted.

### 2.5. DNA Extraction from Bacteria

Total DNA was extracted from bacterial suspensions or plant material using the DNeasy^®^ Blood & Tissue Kit for Gram-positive bacteria (QIAGEN, Hilden, Germany), according to the manufacturer’s instructions. The DNA concentrations were estimated using the NanoDrop 2000c UV-Vis spectrophotometer (Thermo Fisher Scientific Inc., Worchester, MA, USA).

### 2.6. Double-Antibody Sandwich Enzyme-Linked Immunosorbent Assay (DAS-ELISA)

For the ELISA technique, a variant of the sandwich method was used with two different antibodies which recognize different epitopes of the antigene (Reagent Set DAS ELISA, Agdia, Elkhart, IN, USA). The assay was performed according to the manufacturer’s instructions. The measurement of the chromogenic reaction product was carried out by calculating the optical density (OD) of three repetitions for each sample at 405 nm with the iMark™ microplate absorbance reader (Bio-Rad, Hercules, CA, USA).

### 2.7. Immunofluorescence

For the detection of *C. michiganensis* in plant tissues and bacterial suspensions by indirect immunofluorescence staining (IF), rabbit antiserum with polyclonal antibodies were used for primary detection (anti-Clav 25 at 1:1200 dilution, prepared by the Laboratory of Bacteriology of HΜU), while goat anti-rabbit IgG (H + L) conjugated with CF488A dye (Cat No. 20012, Biotium, Fremont, CA, USA) at 1:100 dilution was used as the secondary antibody. Briefly, 15 μL of each sample was placed and heat-fixed on the slides with 10 wells and then incubated with the antiserum for 30 min. The slides were washed three times in 1 × PBS and incubated for 30 min in the dark with the secondary antibody. After the incubation period, the slides were again washed three times in 1 × PBS. Preparations were visualized by fluorescent microscopy with a microscope (Olympus BH-2), Olympus, Tokyo, Japan equipped with the appropriate filters for fluorescein fluorescence at a viewing field of at least 1000× magnification.

### 2.8. Preparation of Plant Samples for LAMP

For the preparation of plant extracts, a transverse section was created in the stems of the seedlings 3 cm above the wounding spot. A thin ring of about 3 mm long was removed from the stem, placed in 500 μL of homogenization solution (TPS buffer:100 mM Tris-HCl, 1 M KCl, 10 mM, EDTA, pH 8), and squeezed thoroughly with a pestle. Part of the extract was diluted (1:5) in dH_2_O, and two μL was used as a template for the LAMP reaction. For the preparation of spiked samples, different concentrations of bacterial suspensions were added to the plant extracts before the dilution, while for infected samples no further treatment on the plant lysate was performed.

### 2.9. LAMP Primer Design and Reaction Conditions

For the development of the LAMP diagnostic tool, the *micA* (michiganin A) gene was used as the target sequence. According to Yasuhara-Bell et al. [13], this sequence is highly specific for *C. michiganensis*. Six primers were designed using the LAMP primer design program (PrimerExplorer) located on the Eiken Chemical Tokyo, Japan, website (http://primerexplorer.jp/e/ (accessed on 10 June 2024)) and are listed in Table 1. These primers were used in two different LAMP set-ups.

For the first set-up, the LAMP reaction was conducted with the use of the portable PEBBLE-R qcLAMP platform [14] and the master mix from the same provider (BIOPIX-T, Heraklion, Crete, Greece). The LAMP reaction had a final volume of 25 μL and contained 1× of the 10× primer mix (CmF3 2 μΜ, CmB3 2 μΜ, CmFIP 18 μΜ, CmBIP 18 μΜ, CmLF 5 μΜ, CmLB 5 μΜ), 1× of the Universal Master Mix (BIOPIX-T, cat.no: 000054) and 2 μL of the template (template’s concentration varies depending on the experiment). To avoid evaporation in the PEBBLE device, 15 μL of mineral oil (included in BIOPIX-T kit) was added in each reaction mixture. The reactions were incubated at 65 °C for 30 min. All LAMP reactions were run on PEBBLE-R qcLAMP platform. PEBBLE platform is a diagnostic system in which the amplification of genetic material molecules and also the real-time monitoring of the amplification through the color change of the samples (quantitative colorimetric LAMP-qcLAMP) is achieved. To enhance and monitor it in real time, the device heats the samples to the appropriate temperature while visualizing the color change through LED lighting and a camera.

For the second set-up, the SuperScriptTM IV RT-LAMP (Thermo Fisher Scientific, Waltham, MA, USA) with fluorescent dye (SYTO 9 green fluorescent nucleic acid stain) was used. The LAMP reaction had a final volume of 25 μL and contained 1× of the 10× concentrated primer mix (CmF3 2 μΜ, CmB3 2 μΜ, CmFIP 18 μΜ, CmBIP 18 μΜ, CmLF 5 μΜ, CmLB 5 μΜ), 1× of the Universal Master Mix, and 2 μL of the template. The reactions were performed on the CFX96 real-time PCR system (Bio-Rad, Hercules, CA, USA) at 65 °C for 60 cycles of 30 s with data collection after each cycle followed by a melting curve.

### 2.10. Polymerase Chain Reaction, PCR

For *C. michiganensis* detection by PCR, the primers were the ones described by the European and Mediterranean Plant Protection Organization (EPPO) [15], PSA-8 and PSA-R, which amplified a 268 bp product. PCR was performed using Platinum Taq DNA polymerase (Invitrogen, Carlsbad, CA, USA) according to the manufacturer’s instructions in the T100™ Thermal Cycler (Bio-Rad, Hercules, CA, USA). The PCR reaction contained 1× buffer, 1.5 mM MgCl_2_, 0.2 mM dNTPs, 0.2 μM of each primer, and 2U Platinum Taq DNA polymerase in a final volume of 25 μL. A total of 2 μL was used as template. After an initial denaturation step at 95 °C for 5 min, each cycle consisted of a 30 s denaturation step at 95 °C, an annealing step at 62 °C, and an extension step for 30 s at 72 °C. The products were analyzed in a 1.7% (*w*/*o*) agarose gel.

### 2.11. Quantitative Polymerase Chain Reaction

The quantitative polymerase chain reaction (qPCR) was performed using TaqMan^®^ Fast Universal PCR Master Mix No AmpErase^®^ UNG (Applied Biosystems, Foster City, CA, USA) according to the manufacturer’s instructions. A 10-fold serial dilution of *C. michiganensins* DNA was used as template. The qPCR reaction contained 1× TaqMan master mix (Applied Biosystems), 2 μM of each primer, and probe and template DNA (varied depending on the experiment) in a final reaction of 20 µL. Each cycle consisted of denaturation at 95 °C for 10 min followed by 40 cycles of: 95 °C for 15 s and 60 °C for 30 s. The primers and probe used were the ones described by the European and Mediterranean Plant Protection Organization (EPPO) (Forward RZ_ptssk 10, Reverse RZ_ptssk 11, and Probe RZ_ptssk) [15]. Reactions were performed on the CFX96 Real-Time PCR System (Bio-Rad, Hercules, CA, USA).

### 2.12. Statistical Analysis

Experiments were conducted in triplicate. Time-to-positive results are reported as the mean ± standard deviation (SD). No complex statistical comparisons were required due to the clear qualitative nature of most readouts (detected/not detected).

## 3. Results

### 3.1. Sensitivity and Specificity of the LAMP Assay

**Figure 1:**
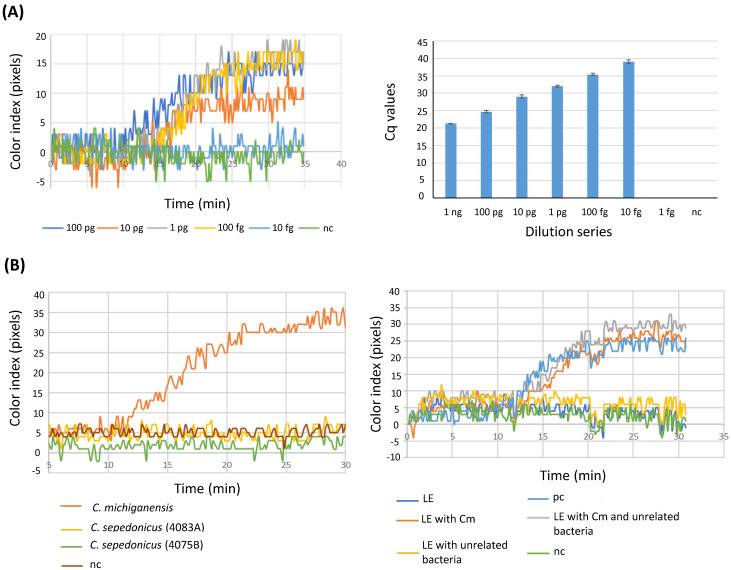
Sensitivity and specificity assessment of the B-LAMP assay. (**A**) Serial dilutions of *C. michiganensis* genomic DNA (100 pg to 10 fg per reaction) analyzed by colorimetric LAMP using the BIOPIX-T platform (left panel) and by qPCR (right panel). (**B**) Specificity analysis of the B-LAMP assay using DNA from *C. sepedonicus* isolates (left panel) and bacterial suspensions of *X. campestris* pv. *vesicatoria*, *P. syringae* pv. *tomato*, and *Bacillus* sp. (right panel). Abbreviations: LE, leaf extract; pc, positive control; nc, negative control.

Initially, the detection limit of the developed LAMP with BIOPIX-T portable device/enzyme mix (from now on referred to as B-LAMP) was determined by testing serial dilutions from extracted bacterial DNA. The same dilution series were also used for the performance of qPCR. As shown in Figure 1A (left panel), the lowest amount of DNA consistently testing positive using the designed LAMP assay was 100 fg per reaction (corresponding to 28 copy numbers) while the detection limit increased one order of magnitude when assessed by qPCR (10 fg/reaction) (Figure 1A, right panel). The specificity of the LAMP assay was tested using DNA from *C. michiganensis* and two isolates of *C. sepedonicus* (Figure 1B, left panel) or bacterial suspensions from *C. michiganensis* and different species of bacteria that are commonly found in tomato plants (*X. campestris* pv. *vesicatoria*, Xcv, *P. syringae* pv. *tomato* (DC3000), and a bacterium belonging to an unidentified *Bacillus* species, GL2, which was isolated from tomato plants) (Figure 1B, right panel). As depicted in Figure 1B, in all cases positive results were produced only in samples containing *C. michiganensis* while the presence of unrelated bacteria had no effect on *C. michiganensis* detection in the sample (Figure 1Β).

### 3.2. Sensitivity of the LAMP Assays in Comparison with Other Detection Methods

**Figure 2:**
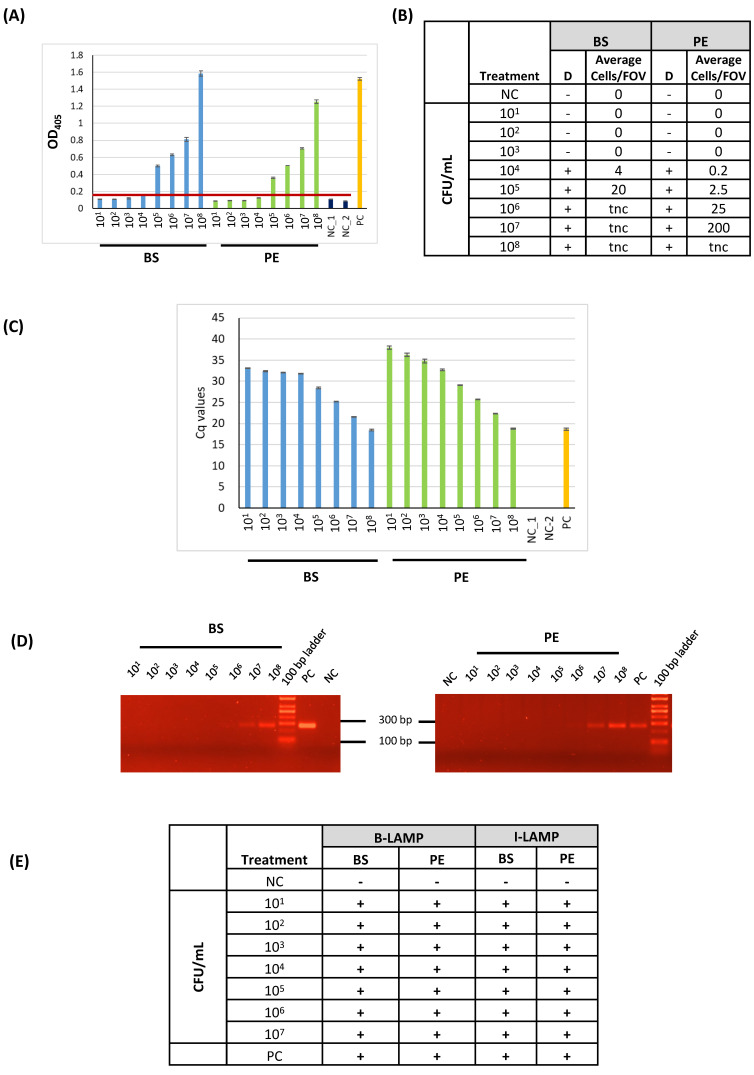
Comparison of detection methods for *C. michiganensis*. Bacterial suspensions (BS) and corresponding plant extracts (PEs) spiked with *C. michiganensis* (10^1^–10^8^ CFU/mL) were analyzed using (**A**) DAS-ELISA, (**Β**) immunofluorescence (IF), (**C**) qPCR, (**D**) PCR, and (**E**) LAMP with the BIOPIX-T system (B-LAMP) or the Invitrogen enzyme mix (I-LAMP). The red line in panel (**A**) indicates the assay threshold. Abbreviations: NC, negative control; PC, positive control; D, detected; tnc, too numerous to count; FOV, field of view.

Next, the LoD of LAMP was compared with other routinely used methods for *C. michiganensis* detection. The experimental procedures compared were the following: (a) LAMP with BIOPIX-T portable device/enzyme mix (B-LAMP), (b) LAMP with the Invitrogen reaction mix (from now on referred to as I-LAMP) (d) DAS-ELISA, (c) immunofluorescence (IF), (d) PCR, and (e) qPCR. For these experiments, the same samples, which were bacterial suspensions ranging from 10^8^ to 10^1^ CFU/mL (termed as BS) or plant extracts (PE) spiked with *C. michiganensis* of different concentrations (10^8^ to 10^1^ CFU/mL), were compared. For *C. michiganensis* detection with the molecular methods (LAMP assays, PCR, and qPCR), DNA was extracted from each sample, eluted in an equal volume of elution buffer, and the same volume (2 μL) was used as a template in each method. As depicted in Figure 2, the LoD varied depending on the initial sample used (ΒS vs. PE) and the method of detection. In more detail, qPCR (Figure 2C), B-LAMP, and I-LAMP (Figure 2E) were able to detect *C. michiganensis* in all samples, reaching an LoD of 10^1^ CFU/mL. PCR (Figure 2D) reached a marginal LoD in both treatments (BS and PE) of 10^6^ CFU/mL, while DAS-ELISA (Figure 2A and Supplementary Figure S1) had an LoD of 10^5^ CFU/mL and, lastly, IF (Figure 2B) had a reliable LoD of 10^4^ in BS samples and an LoD of 10^5^ in PE samples. Altogether, the LAMP assays had an equal LoD with the most sensitive method, qPCR, when DNA was extracted from the samples. The LοD using purified genomic DNA (100 fg/reaction, ≈28 genome copies; Section 3.1) was comparable with the LOD achieved after DNA extraction from bacterial suspensions or spiked plant extracts (10^1^ CFU/mL; Section 3.2), indicating that the DNA extraction step introduced a minor reduction in overall assay sensitivity.

### 3.3. Evaluation of the LoD of C. michiganensis-LAMP Assay in Lysis Buffer and Plant Extracts Spiked with the Pathogen

Since the development of this portable *C. michiganensis*-LAMP assay aims at detecting *C. michiganensis* in plant tissues with minimum sample processing, the LoD was compared between plant extracts from the stems of tomato seedlings vs. plain lysis buffer, both spiked with different *C. michiganensis* concentrations (10^6^–10^4^ CFU/mL). In all cases, the samples were used without DNA extraction but directly with a one-fifths dilution in water. The results showed that the presence of plant extracts negatively affected the LoD by an order of magnitude (Table 2). In more detail, the minimal bacterial load that could be detected in lysis buffer spiked with *C. michiganensis* was 10^4^ CFU/mL while in those samples containing plant extracts the LoD corresponded to 10^5^ CFU/mL. Additionally, the potential inhibitory effect of plant material was evaluated by conducting separate experiments using increased amounts of plant tissue (5× more, corresponding to 0.5 g/mL lysis buffer) in spiked extracts (Table 3). Spiked samples denser in plant extracts were documented as false negative in the *C. michiganensis*-LAMP assay, an effect that could be overcome by increasing the dilution factor of the sample in water to 1/25. After this increase in sample dilution, the *C. michiganensis*-LAMP assay could again detect the pathogen at a concentration of 10^5^ CFU/mL (Table 3). Thus, increasing the amount of plant tissue in the lysate does not improve the LoD because the excess of plant metabolites impedes the enzymatic process, resulting in false negatives in the LAMP assay.

### 3.4. Assessment of the Effect of Plant Extracts from Different Tomato Cultivars

In agriculture, several tomato cultivars are grown based on the farmer/consumer’s preferences. However, each one may have a unique metabolic profile, which might potentially hinder downstream enzymatic reactions, such as the LAMP assay. To validate the efficacy of *C. michiganensis*-LAMP in the pathogen’s detection independent of the tomato cultivar used, eight commercial tomato cultivars that are used locally were examined: Lobello, Kalloni, Ekstasi, Lesvos, Elpida, Christina, Nissos, and Aethra. The stems of the tomato seedlings were used for the preparation of the plant extracts and were spiked with *C. michiganensis* suspension (10^5^ CFU/mL). After the appropriate one-fifths dilution of the spiked extract in water, the samples served as templates in the LAMP assay. The pathogen was effectively detected regardless of the tomato variety that was analyzed (Table 4), suggesting that different metabolic profiles of tomato cultivars do not affect the pathogen’s detection by LAMP.

### 3.5. Validating C. michiganensis Detection in Experimentally Inoculated Tomato Plants

The LAMP assay was validated in tomato plants that were experimentally inoculated with *C. michiganensis* at different time points from the inoculation day (4, 7, and 12 days post-inoculation, dpi). In each case, the plant extracts were prepared from the stem tissue 3 cm above the point of inoculation and after one-fifths dilution. In parallel, plant extracts were used to assess the bacterial population. As shown in Table 5, when the bacterial population in the plant extracts was lower than the LoD, the pathogen could not be detected. In all other cases, even 4 dpi, the B-LAMP assay could readily detect the pathogen, even though the plants were still asymptomatic.

## 4. Discussion

Effective disease management strategies cannot be implemented without the availability of sensitive, reliable, rapid, and affordable detection techniques. Apart from the traditional pathogenicity tests used to identify *C. michiganensis*, various papers have been reported that collectively showcase a range of other detection methods—molecular (PCR, qPCR) [16,17,18,19,20,21], immunological or with immunomagnetic separation [11], and spectroscopic (Raman) [22], offering options depending on sensitivity, speed, and sample-type needs. Despite the sensitivity some of them are offering, they are coupled with inherent disadvantages such as the need for centralized laboratories, specialized personnel, and equipment, to name a few. LAMP-based detection assays, on the other hand, developed for *C. michiganensis*’s detection [13,23,24] offer an option for field-based applications since portable devices are now available. Since the LAMP-related studies for the pathogen’s detection relied on laboratory equipment, the aim of this study was to develop and evaluate the efficiency of field-based *C. michiganensis* detection with the use of portable LAMP devices.

Initially, the sensitivity of the designed primer set was validated. The primers target the *micA* gene, which codes for an antimicrobial peptide, *Michiganin* A, produced by the pathogen *C. michiganensis* and which was previously reported to be *C. michiganensis*-specific [23]. From the colorimetric real-time LAMP assay using the portable device PEBBLE (BIOPIX-T), it was possible to detect up to 100 fg per reaction of *C. michiganensis*-derived DNA corresponding to 28 copies based on genome size, while qPCR could detect up to 10 fg of bacterial DNA. Comparing the LoD in this study, when pure bacterial DNA was used as the template, with the corresponding LoD from other studies using LAMP for detecting plant pathogenic bacteria [25,26,27,28], a higher sensitivity in the detection limit was achieved. A higher LoD (1 fg per reaction) was documented by Dobhal et al. [24], but they did not use species-specific primers.

Concerning the validation of the detection limit of B-LAMP against other routinely used methods (ELISA, IF, qPCR, and PCR), B-LAMP had an equal LoD with qPCR (10^1^ CFU/mL) and the same performance when using I-LAMP, which was implemented in laboratory equipment as opposed to the portable device used in B-LAMP. PCR, on the other hand, could marginally detect 10^6^ CFU/mL. The marked difference in sensitivity, where PCR exhibited an LoD approximately 10,000-fold lower than qPCR, stems primarily from differences in detection methodology. While the PCR relied on standard *Taq* polymerase and less sensitive endpoint detection by gel electrophoresis, the TaqMan qPCR utilizes real-time fluorescence monitoring and probe-based chemistry optimized for high analytical sensitivity.

DAS-ELISA and IF could reliably detect a concentration of 10^5^ CFU/mL in plant extracts. Regarding the LoD from bacterial suspensions, there have been reports of detecting as little as 10^2^ CFU/mL [27] or 10^3^ CFU/mL [26] when DNA extraction from samples were performed by a quick or standard method. Thus, the B-LAMP method developed in this study has a higher sensitivity when DNA is extracted from the samples.

Yasuhara et al. [13] have thoroughly tested the *micA* gene primers for specificity towards *C. michiganensis*. Here, the same gene was used to design primers for LAMP, which were evaluated against bacteria belonging to the same genus (*C. sepedonicus*) or different genera (*Xanthomonas*, *Pseudomonas*, and *Bacillus* sp.) No false positives were scored in any of the cases.

The aim of this study was to develop and assess a LAMP assay that will require the fewest treatments possible, allowing non-specialized personnel to utilize it using simple tools and reagents. The LoD when plant extracts were spiked with *C. michiganensis* was an order of magnitude lower (10^5^ CFU/mL) than the LoD observed in the corresponding bacterial suspension (10^4^ CFU/mL). This is expected since the metabolites of the plants can impede downstream enzymatic reactions like the LAMP assay. With respect to this, the method was evaluated in plant extracts from eight tomato varieties and found no impact in the detection sensitivity. Of course, the goal of all detection protocols is directed to pathogen detection in infected plant tissues per se. In this aspect, the method was applied in experimentally inoculated tomato seedling and could detect the pathogen as early as four dpi. In each case, inoculated samples with lower bacterial populations than the LoD could not be scored as positive.

Considering the limitations of serological techniques and qPCR due to the requirement for specialized staff and equipment, *C. michiganensis*-LAMP appears to be a reliable and precise technique for first disease assessment in the field, regardless of the tomato variety. It represents a user-friendly detection method and a valuable alternative for on-site disease assessment. The developed cLAMP assay proved robust under the controlled and semi-field conditions tested but broader field validation across diverse growth stages, and environmental conditions will be essential to be tested to confirm its reliability for routine diagnostic use. Lastly, its sensitivity can be increased if DNA is extracted from the samples.

## Figures and Tables

**Table 1:** Loop-mediated amplification (LAMP) primers used in this study.

Primer Name	Sequence (5′–3′)
CmF3	CCATGCGCGACAACAGG
CmB3	ACATGTACGGGCTCACGA
CmFIP	GGCTGACCATGACGGGGGTCAACACAGGTGGAACACGATG
CmBIP	CGGTGGGACATGCTGCTCGACTCCGCGTCGATCTGG
CmLF	TCCGTCTCGAGGATGTCGTT
CmLB	GGGCGAGGACACCAGCCCGT

**Table 2:** Comparison of the LoD of the LAMP assay between the suspension buffer and plant extracts spiked with *C. michiganensis*. Bacterial suspensions of various concentrations were used to spike lysis buffer or plant extracts and served as templates for LAMP after a 1/5 dilution in dH_2_O. n/d = not detected.

Treatment	Number of Replications	Minutes to Result (Means)	SD
Plant extract spiked with 10^6^ CFU/mL	4	15.5	0.5
Plant extract spiked with 10^5^ CFU/mL	4	20.5	0.5
Plant extract spiked with 10^4^ CFU/mL	4	n/d	
Bacterial suspension 10^6^ CFU/mL	4	15.4	0.1
Bacterial suspension 10^5^ CFU/mL	4	18.15	1.15
Bacterial suspension 10^4^ CFU/mL	4	23.95	0.45
Negative control	6	n/d	
Positive control	6	12.5	0.38

**Table 3:** Adverse impact in *C. michiganensis* detection by LAMP when an increased amount of plant tissue (5×) is used for the preparation of the samples. An amount of 5× more plant tissue (0.5 g/mL of suspension buffer) was used for the preparation of the samples, resulting in false negatives. n/d, not detected.

Treatment	Number of Replications	Minutes to Result (Means)	SD
Plant extract spiked with 10^6^ CFU/mL	4	16.5	1.5
Plant extract spiked with 10^5^ CFU/mL	4	n/d	
Plant extract spiked with 10^4^ CFU/mL	4	n/d	
Additional dilution of plant extract spiked with 10^6^ CFU/mL	4	14.7	0.5
Additional dilution of plant extract spiked with 10^5^ CFU/mL	4	19.3	0.8
Additional dilution of plant extract spiked with 10^4^ CFU/mL	4	n/d	
Negative control	4	n/d	
Positive control	4	12.7	0.28

**Table 4:** Detection of *C. michiganensis* by the B-LAMP assay in eight different tomato commercial varieties. All samples were spiked with the same concentration of the pathogen (10^5^ CFU/mL). * n/d = not detected.

Extracts from Different Tomato Cultivars	Minutes to Positive Result
Lobello—spiked	17.5
Kalloni—spiked	15.7
Ekstasis—spiked	16.5
Lesvos—spiked	17.3
Elpida—spiked	18.7
Christina—spiked	16.0
Nissos—spiked	14.7
Aethra—spiked	14.5
Lobello—control	n/d *
Kalloni—control	n/d
Ekstasis—control	n/d
Lesvos—control	n/d
Elpida—control	n/d
Christina—control	n/d
Nissos—control	n/d
Aethra—control	n/d
Cm gdna	12.7

**Table 5:** *C. michiganensis* detection in experimentally inoculated tomato plants by the B-LAMP assay. * n/d = not detected.

dpi	Sample Name	Plant Number	Minutes to Result	Cell Number per 1 mL	Cell Number in LAMP Reaction
4	Plant extracts from healthy plants	1	n/d *	0	0
		2	n/d	0	0
		3	n/d	0	0
		4	n/d	0	0
	Plant extracts from inoculated plants	1	n/d	133	0
		2	14.2	7.3 × 10^7^	2.93 × 10^4^
		3	14.8	4.47 × 10^6^	1.79 × 10^3^
		4	n/d	1.73 × 10^4^	6.93 × 10^0^
7	Plant extracts from healthy plants	1	n/d	0	0
		2	n/d	0	0
		3	n/d	0	0
		4	n/d	0	0
	Plant extracts from inoculated plants	1	n/d	0	0
		2	n/d	0	0
		3	12.8	6 × 10^7^	2.40 × 10^4^
		4	12.5	3.1 × 10^7^	1.25 × 10^4^
12	Plant extracts from healthy plants	1	n/d	0	0
		2	n/d	0	0
		3	n/d	0	0
		4	n/d	0	0
	Plant extracts from inoculated plants	1	15.3	5.1 × 10^6^	2.15 × 10^3^
		2	13.7	5.1 × 10^7^	2.05 × 10^4^
		3	13.3	5.5 × 10^7^	2.21 × 10^4^
	Positive control (Cm gDNA)		12.5		

## Data Availability

The original contributions presented in this study are included in the article/Supplementary Materials. Further inquiries can be directed to the corresponding authors.

## Supplementary Materials

The following supporting information can be downloaded at: https://www.mdpi.com/article/10.3390/plants15030372/s1. Figure S1. Representative photograph of DAS-ELISA microplate showing color development for serial dilutions of *C. michiganensis* in bacterial suspensions (BS) and plant extracts (PE). Wells with stronger yellow color indicate higher bacterial concentrations. Abbreviations: PC, positive control; NC, negative control.
