# Supplementary figures and images for: Development and Validation of a Field-Based Colorimetric LAMP Assay for the Detection of Clavibacter michiganensis in Tomato Plants
Source: Plants (Basel). 2026 Jan 25;15(3):372. doi: 10.3390/plants15030372 (PMC12899487; doi:10.3390/plants15030372)

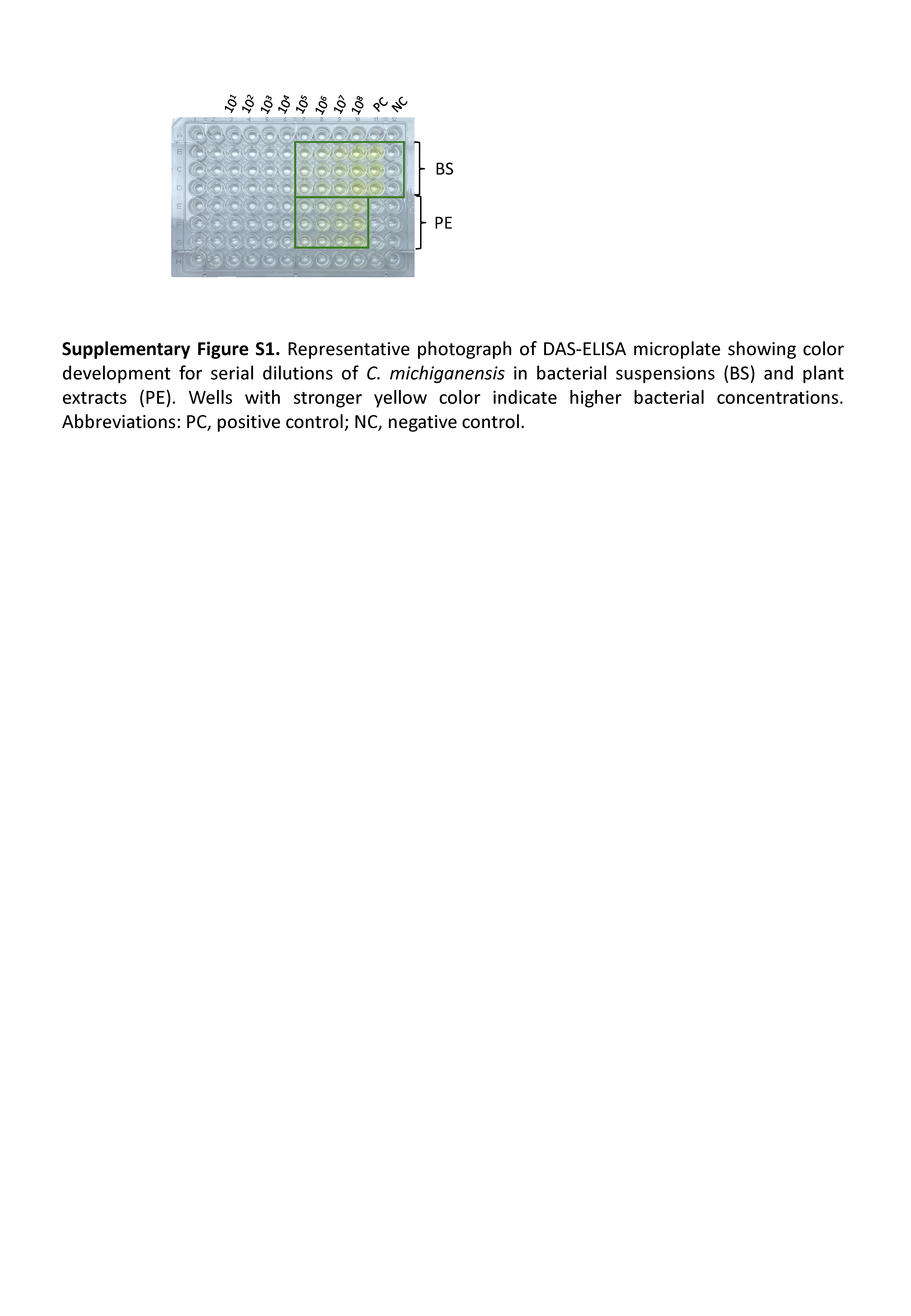

Supplement: Supplementary file 1 [file plants-15-00372-s001.zip › Supplementary Figure S1.tiff]
